# Emotional Competency in Education: Special Issue on Emotional Intelligence and Creativity

**DOI:** 10.3390/jintelligence12060060

**Published:** 2024-06-07

**Authors:** Macarena-Paz Celume, Franck Zenasni

**Affiliations:** 1 Centre de Recherche en Psychologie de la Connaissance, du Langage et de l’Émotion (Centre PsyCLE, EA 3273), Institut Créativité et Innovations, Université Aix-Marseille, 13385 Marseille, France; 2 LaPEA, Université Paris Cité and Univ Gustave Eiffel, 92100 Boulogne-Billancourt, France; franck.zenasni@u-paris.fr

According to Salovey and Mayer (1990), having high EI allows individuals to think clearly, supports intuition and insight, and ultimately enhances creative thinking. Studies have since explored the relationship between EI and creativity, revealing significant but intricate connections. Early research by Ivcevic et al. (2007) and Zenasni and Lubart (2008) indicated that the link between EI and creativity could vary based on how creativity is measured and the type of task used to assess EI. Subsequent studies, including the review conducted by Xu et al. (2021), confirmed moderate relationships between EI and creativity. However, these relationships are influenced by factors such as the specific measures employed for EI and creativity.

Most data refer to EI and creativity in adults, with few studies examining these aspects in children, particularly in an educational context. The limited studies that address this relationship focus on emotions or affect in play and creative thinking (Russ and Kaugars 2001), or mood and creative thinking (Celume et al. 2023).

Despite some empirical evidence, it is insufficient to fully explain the diversity and complexity of the emotional intelligence–creativity relationship in education. Moreover, there is no comprehensive theoretical model that clearly articulates the interplay between the emotional world of children (either as emotional intelligence, competencies, mood, or skills) and their creativity. In this sense, further collaborative research is needed, necessitating this Special Issue.

We have successfully gathered original studies that combine the main concepts of creativity and emotional intelligence, as well as related concepts such as emotional competencies, socio-emotional learning, and psychosocial competencies. The broad scope of this issue includes submissions addressing the development of both creativity and emotional competencies through training programs in education, as explained by Maoulida et al. (2023), who developed an online program based on the 21st century competencies. It also addresses the influences of emotional intelligence on creativity among adults, such as in the study by Puente-Díaz (2023), who explored how the use of metacognitive feelings can be used to assess and select creative ideas. Moreover, theoretical links are established between these constructs in general population, such as in Sundquist and Lubart’s study (2022), which explores these relationships using the model of the seven Cs of Creativity as a structuring framework.

Some of the submissions, like the study of Eschenauer et al. (2023), discuss the complexity and dynamics of language as a cognitive process within the enactive paradigm. Their study investigates how performative theatre can synergistically stimulate creativity, emotional skills, and executive functions, especially in the context of foreign language teaching for children with communication disorders. Their CELAVIE pilot study reveals progress in oral communication, emotional skills, and creativity for a pupil with a neurodevelopmental disorder.

From a more cognitive perspective, Agnoli et al. (2023) explores the modulatory role of trait emotional intelligence on children’s creative potential, examining the influence of cognitive resources and contextual–environmental factors. Their studies highlight the importance of trait EI in enhancing the positive effects of executive functions and teachers’ conceptions of creativity on children’s creative performance, with implications for understanding trait EI as a key component of children’s creativity.

The study by Hoffmann et al. (2023) evaluates the inspirED program, designed to bolster skills valued by employers, such as problem-solving, self-management, and working with people. Their study shows that inspirED projects led to improvements in school climate and student skills, including purpose, self-awareness, and emotional safety, with potential implications for future school-based social–emotional learning and student leadership opportunities. Meanwhile, Sanchez and Blanc (2023) examined differences between gifted and non-gifted children, finding higher scores for creativity among gifted children, along with a gap in socio-affective development.

Botella et al. (2022) investigated the creative process in a real educational context. Their study of Swiss pupils engaged in creative and manual activities provides a model describing the stages and factors involved in the creative process, emphasizing the importance of ecological validity in research on creativity in education.

Finally, Garaigordobil et al. (2022) presents results from four cooperative–creative game programs, demonstrating significant impacts on social, emotional, and cognitive development in children. Their findings underscore the relevance of cooperative–creative play in enhancing various positive behaviors, emotional stability, and creativity, providing empirical evidence for its role in child development.

These contributions collectively enhance our understanding of the multifaceted relationship between emotional intelligence, emotional competencies, and creativity, offering valuable insights for both research and practical applications in educational settings. In today’s educational landscape, there is growing recognition of the importance of nurturing psychosocial competencies that encompass not just cognitive abilities but also social and emotional competencies. By delving into the intricate interplay between various dimensions of human behavior and cognition, these findings provide educators, policymakers, and researchers with nuanced insights into fostering a more comprehensive approach to student development.

In an era marked by rapid technological advancements and societal shifts, the cultivation of psychosocial competencies is increasingly recognized as essential for preparing students to thrive in diverse and ever-changing environments. These competencies, which encompass emotional intelligence, interpersonal skills, resilience, and adaptability, are integral not only to academic success but also to overall well-being and success in life. By incorporating insights from research on emotional intelligence, emotional competencies, and creativity into educational practices, educators can better equip students with the tools they need to navigate the complexities of the modern world with confidence and resilience.

Moreover, by emphasizing the development of these multifaceted competencies in the classroom, educators can contribute to the creation of more inclusive and supportive learning environments. Students who are proficient in emotional competencies and interpersonal skills are better equipped to collaborate effectively, communicate empathetically, and navigate conflicts constructively. This not only enhances their academic performance but also fosters a sense of belonging and community within the classroom.

Ultimately, the integration of research findings on emotional intelligence, emotional competencies, and creativity into educational practices has the potential to yield profound benefits for individuals, communities, and society as a whole. By prioritizing the cultivation of psychosocial competencies alongside academic skills, educators can empower students to become lifelong learners who are capable of adapting to and thriving in an ever-changing world.

## References

[B1-jintelligence-12-00060] Agnoli Sergio, Mastria Serena, Mancini Giacomo, Corazza Giovanni, Franchin Laura, Pozzoli Tiziana (2023). The Dynamic Interplay of Affective, Cognitive and Contextual Resources on Children’s Creative Potential: The Modulatory Role of Trait Emotional Intelligence. Journal of Intelligence.

[B2-jintelligence-12-00060] Botella Marion, Didier John, Lambert Marie-Dominique, Attanasio Rachel (2022). The Creative Process and Emotions of Pupils in a Training Context with a Design Project. Journal of Intelligence.

[B3-jintelligence-12-00060] Celume Macarena-Paz, Ivcevic Zorana, Zenasni Franck (2023). Mood and creativity in children: Differential impacts on convergent and divergent thinking. Psychology of Aesthetics, Creativity, and the Arts.

[B4-jintelligence-12-00060] Eschenauer Sandrine, Tsao Raphaele, Legou Thierry, Tellier Marion, André Carine, Brugnoli Isabelle, Tortel Anne, Pasquier Aurélie (2023). Performing for Better Communication: Creativity, Cognitive-Emotional Skills and Embodied Language in Primary Schools. Journal of Intelligence.

[B5-jintelligence-12-00060] Garaigordobil Maite, Berrueco Laura, Celume Macarena-Paz (2022). Developing Children’s Creativity and Social-Emotional Competencies through Play: Summary of Twenty Years of Findings of the Evidence-Based Interventions “Game Program”. Journal of Intelligence.

[B6-jintelligence-12-00060] Hoffmann Jessica, France Kalee De, McGarry Julie (2023). Creativity and Connection: The Impact of inspirED with Secondary School Students. Journal of Intelligence.

[B7-jintelligence-12-00060] Ivcevic Zorana, Brackett Marc, Mayer John (2007). Emotional Intelligence and Emotional Creativity. Journal of Personality.

[B8-jintelligence-12-00060] Maoulida Haifat, Madhukar Manisha, Celume Macarena-Paz (2023). A Case Study of 21st Century Cognitive, Social and Emotional Competencies Using Online-Learning. Journal of Intelligence.

[B9-jintelligence-12-00060] Puente-Díaz Rogelio (2023). Metacognitive Feelings as a Source of Information for the Creative Process: A Conceptual Exploration. Journal of Intelligence.

[B10-jintelligence-12-00060] Russ Sandra, Kaugars Astrida (2001). Emotion in children’s play and creative problem solving. Creativity Research Journal.

[B11-jintelligence-12-00060] Salovey Peter, Mayer John (1990). Emotional Intelligence. Imagination, Cognition and Personality.

[B12-jintelligence-12-00060] Sanchez Christine, Blanc Nathalie (2023). Abstract Graphic Creativity, Feelings about School, and Engagement in the School Environment: What Are the Interindividual Differences between Gifted and Non-Gifted Children?. Journal of Intelligence.

[B13-jintelligence-12-00060] Sundquist Daniel, Lubart Todd (2022). Being Intelligent with Emotions to Benefit Creativity: Emotion across the Seven Cs of Creativity. Journal of Intelligence.

[B14-jintelligence-12-00060] Xu Xiaobo, Pang Weiguo, Xia Mengya (2021). Are emotionally intelligent people happier ? A meta-analysis of the relationship between emotional intelligence and subjective well-being using Chinese samples. Asian Journal of Social Psychology.

[B15-jintelligence-12-00060] Zenasni Franck, Lubart Todd (2008). Emotion-related traits moderate the impact of emotional state on creative performances. Journal of Individual Differences.

